# COA6 promotes the oncogenesis and progression of breast cancer by oxidative phosphorylation pathway

**DOI:** 10.7150/jca.98570

**Published:** 2024-08-01

**Authors:** Xiaoxia Jin, Xinyan Chen, Haiyan Yu, Yushan Liu, Xiaoyun Lu, Haibing Yin, Wencheng Dai

**Affiliations:** 1 Department of Pathology, Nantong Tumor Hospital, Affiliated Tumor Hospital of Nantong University, Nantong, Jiangsu, China.; 2 Department of Head and Neck Surgery, Nantong Tumor Hospital, Affiliated Tumor Hospital of Nantong University, Nantong, Jiangsu, China.; 3 Medical School of Nantong University, Nantong, Jiangsu, China.

**Keywords:** COA6, Breast cancer, OXPHOS, The Cancer Genome Atlas (TCGA)

## Abstract

Mitochondrial oxidative phosphorylation (OXPHOS) has long been considered the primary energy source in breast cancer cells. Cytochrome c oxidase assembly factor 6 (COA6), which functions as a metal chaperone to transport copper to complex Ⅳ during the OXPHOS process, plays a crucial role in the carcinogenesis of lung adenocarcinoma. Nevertheless, its specific function in breast cancer is undefined. The present investigation aimed to clarify COA6's expression profile and regulatory functions in breast cancer, as well as to unveil its underlying mechanisms. Initially, our findings revealed a significant upregulation of COA6 in breast cancer, as evidenced by an analysis of the TCGA database and tissue microarrays. This upregulation correlated with tumor size and histological grade. Additionally, survival analysis revealed that elevated COA6 amounts were correlated with decreased overall survival (OS) in breast cancer. To delve deeper into the functions of COA6, both COA6-overexpressing and COA6-knockdown breast cancer cell models were established. These experiments demonstrated COA6 is pivotal in regulating cell proliferation, apoptosis, migration, and invasion, thereby promoting cancer progression *in vitro*. Notably, functional enrichment analysis indicated COA6 might be involved in breast cancer progression by modulating oxidative phosphorylation (OXPHOS). Collectively, this study reveals an overt tumorigenic role for COA6 in breast cancer and sheds light on its potential mechanisms, offering valuable therapeutic targets for breast cancer therapy.

## Introduction

The Global Cancer Report 2020 revealed a startling statistic: breast cancer has become the most prevalent malignant disease worldwide, surpassing lung cancer for the first time. Breast cancer has a particularly high mortality rate among women, causing over 685,000 deaths 1. While standard treatment regimens typically combine surgical intervention with radiotherapy, endocrine therapy, and/or chemotherapy, such approaches are not always efficient, especially when patients develop resistance to chemotherapeutic agents 2. This underscores the urgent need to unveil the mechanisms driving breast cancer progression, which may help develop new therapeutic targets.

Historically, the function of oxidative phosphorylation (OXPHOS) in tumors has been mostly overlooked. According to the "Warburg effect" theory, tumor cells primarily rely on glycolytic energy production, with suppressed OXPHOS pathway, even with adequate oxygen 3. However, recent advances in tumor metabolism research have provided compelling evidence that OXPHOS serves as the primary energy source for multiple malignancies, including leukemia, lymphoma, pancreatic ductal adenocarcinoma, malignant melanoma, endometrial cancer, and glioma 4,5. It is worth noting that breast cancer also shows pronounced OXPHOS activity. At the Shanghai Cancer Center of Fudan University, a thorough assessment of the global metabolic transcriptional profile of triple-negative breast cancer revealed OXPHOS and pyrimidine metabolism as the most upregulated metabolic pathways 6.

Cytochrome c oxidase assembly factor 6 (COA6) plays a crucial role in electron transport during OXPHOS. As a copper chaperone, COA6 is responsible for delivering copper to complex Ⅳ, which is an essential component of the electron transport chain 7-9. Evidence suggests that COA6 deletions or mutations can suppress the assembly of complex Ⅳ, resulting in multiple mitochondria-related disorders, including fatal cardiomyopathy in infants 10,11. However, the associations of COA6 with tumors remain uncertain, with only a few reports directly linking COA6 to tumorigenesis. According to previous studies, Adrb2 deletion in endothelial cells increases COA6 expression, enhances OXPHOS, and promotes prostate cancer progression 12. Similarly, Zhang and collaborators developed a risk model associating high COA6 expression with poor prognosis in lung adenocarcinoma 13. However, no previous report has examined the association of COA6 with breast cancer.

In the current study, whole transcriptome sequencing was performed to identify differentially expressed genes, focusing on those associated with OXPHOS. COA6 is considered a significant player among these genes, with its mRNA levels notably elevated in breast cancer tissues and this overexpression directly impacting patient survival. Immunohistochemical (IHC) analysis further investigated the protein expression and clinical relevance of COA6 in breast cancer. Furthermore, we demonstrated a causal role for COA6 in the regulation of biological behaviors of breast cancer cells by establishing stable cell lines with overexpressed and silenced COA6. Mechanistically, it was found that high COA6 expression stimulates OXPHOS, providing an energy boost for tumor cell growth. Overall, the above data indicate COA6 could serve as a promising therapeutic target in clinical breast cancer.

## Materials and Methods

### Patient data and sample collection

For comprehensive transcriptome sequencing analysis, 12 breast infiltrating ductal cancer cases were selected, while 125 breast infiltrating ductal cancer cases were examined by IHC analysis. All patients, enrolled in the Affiliated Tumor Hospital of Nantong University, did not receive any anti-tumor therapy before surgery. Tumor tissue samples, surgically excised, were immediately stored at -80°C, and pathological paraffin sections were obtained at the hospital's Pathology Department. All samples were pathologically diagnosed based on the World Health Organization (WHO) classification, and were referred to the protocol approved by the Ethics Committee of the Affiliated Tumor Hospital of Nantong University (Ethical Approval Number: 2022-002).

### Acquisition and maintenance of cell lines

The Cell Culture Collection of the Chinese Academy of Sciences (Shanghai, China) provided four breast cancer (MDA-MB-231, HCC1937, MCF7, SK-BR-3) and one benign breast (MCF-10A) cell lines. The culture specifics were as follows: MDA-MB-231 and MCF-7 cells were cultured in Dulbecco's modified Eagle's medium (DMEM, Gibco, USA) containing 10% fetal bovine serum (FBS, Gibco). HCC1937 and SK-BR-3 cells underwent culture in RPMI 1640 medium (Gibco) also containing 10% FBS. DMEM/F-12 medium (1:1) containing 10% FBS was utilized to culture MCF-10A cells. The cell cultures were maintained in an incubator at 37°C and 5% CO_2_.

### The Cancer Genome Atlas (TCGA) database mining

Differentially expressed genes (DEGs) in breast cancer were retrieved from TCGA (https://tcga-data.nci.nih.gov/) using stringent criteria. Only genes with a log2FC greater than 0 and *P*<0.0001 were considered DEGs.

### Lentiviral acquisition and cellular transfection

Specialized lentiviruses expressing various forms of COA6 (overexpression COA6, shCOA6-1, and shCOA6-2) along with corresponding control vectors were provided by GeneChem (Shanghai, China). For lentivirus transfection, MCF7 cells were transduced with overexpression of COA6 lentivirus at the infection MOI≥90, while MDA-MB-231 and HCC1937 cells underwent shCOA6 lentivirals transfection at an MOI≥50 for at least 24 h. Then cells were cultured in the medium contained 10% FBS and continuously selected with 2 μg/mL puromycin G418 (Invitrogen, USA). The transfection efficiency was rigorously assessed post-selection by qRT-PCR and immunoblot.

### Immunohistochemistry staining and score

Paraffin-embedded sections were examined using an IHC protocol that involved xylene dewaxing, and rehydration via an ethanol gradient. Upon antigen retrieval using 0.1 M citrate buffer boiling for 30 min at 100℃, 3% H_2_O_2_ was employed to block endogenous peroxidase. Primary antibody anti COA6 (1:100) was incubated for 2 h at 37℃, followed by washes and incubation with a secondary antibody at the room temperature. For chromogen development, the Dako Envision system/HRP (Dako Cytomation, Denmark) was used. Freshly prepared DAB was employed, followed by hematoxylin counterstaining. Finally, dehydration, treatment with xylene, and mounting (neutral gum) were performed for each section.

According to the evaluation criteria of the WHO classification, cells stained with nuclei were classified as negative staining (-), low (<20% positive), medium (20%-50% positive), and high (>50% positive). The expression of cytoplasmic staining was divided into negative staining (-), low (<25% positive), medium (25%-50% positive) and high (>50% positive) groups. The staining intensity was scored as 0, negative; 1, weak; 2, medium; 3, strong. 0-1 were classified as low expression and 2-3 as high expression.

### RNA extraction and qRT-PCR

RNA extraction, reverse transcription, and qRT-PCR followed the manufacturer's protocols. Key reagents, including the TransZol Up Plus RNA Kit, the All-in-One First-Strand cDNA Synthesis SuperMix and the Green qPCR SuperMix, were from Transgen Biotech (Beijing, China). Amplification in qRT-PCR was carried out using specific primer sequences: COA6, sense 5'-GCCTGCGTGACAGTTTCCTC-3' and antisense 5'-TGCCATTCTACTGCGATGA-3'; ACTB, sense 5'-GCGTGACATTAAGGAGAAGC-3' and antisense 5'-CCACGTCACACTTCATGTGG-3'.

### Western blot analysis

Total protein extraction was lysed in Radioimmunoassay Lysis Buffer (ThermoFisher Scientific, USA) directly and determined the concentration by BCA Protein Assay Kit (Beyotime Biotechnology, Beijing, China). Western blot analysis followed standard protocols: about 30 μg total protein was separated by 10% SDS-PAGE and then transferred onto the PVDF membrane (Millipore, Danvers, MA, USA). After blocked by 5% skim milk, the membrane was incubated with primary antibodies targeting COA6 (1:1000), NDUFS3 (1:1000), NDUFA4 (1:1000), COX5A (1:1000), UQCRFS1 (1:1000), ATP5G1 (1:1000), β-actin (1:2000), GAPDH (1:2000) at 4℃overnight. The next day, the membrane was washed with TBS-T buffer and then incubated with appropriate secondary antibodies at 37℃ for 2 h. Finally, the samples were detected by the ECL system (ThermoFisher Scientific).

### Celigo cell counting

Stably transfected cells at 2000/well were seeded in a 96-well plate. Starting on day 2 post-seeding, the Celigo system was utilized for daily cell count analysis over a 5-day period. Precise determination of fluorescent cell numbers was achieved via adjusted analysis settings, with subsequent graphing to generate a proliferative curve.

### Clonogenic assay

Cells (2000/well) after transfection were seeded in a 6-well plate. Colonies were visualized by crystal violet staining and enumerated after 14 days of incubation.

### MTT assay

Cells (2000/well) after transfection were seeded in a 96-well plate. At 24, 48, 72, and 96 h, the MTT solution was added for 4 h. Cell viability was assessed by absorbance reading at 490 nm, which served as a proxy for metabolic activity.

### Cell cycle analysis

Cell cycle progression was examined flow-cytometrically employing a cell cycle analysis kit (Beyotime Biotechnology). Stably infected cells underwent fixation with 70% alcohol for 3 h at 4°C, three PBS washes, and centrifugation (1,000 g for 5 min). The resulting cell pellets were incubated in 500 μL PI solution for 30 min. Cell cycle distribution was assessed on a BD FACS ARIA II flow cytometer (Becton Dickinson, USA).

### Cell apoptosis assay

Cellular apoptosis was evaluated using the Annexin-V-FITC kit (Becton Dickinson). Stably transfected cells underwent a 5-minute centrifugation at 200 g, and the pellets were resuspended in binding buffer at ambient to 10^6^ cells/mL. Then, Annexin-V-FITC and PI (5 μL each) were added to a 100-μL aliquot, followed by a 15-minute incubation in the dark at ambient. Then, 400 μL of binding buffer was added, and a dual laser FACS VantageSE flow cytometer (Becton Dickinson) was employed for analysis. Approximately 10,000 cells per assay were analyzed. The percentage of apoptotic cells was then calculated.

### Cell scratch assay

For the scratch assay, cells after stable transfection were seeded into 96-well plates. Once cell confluence approached 90% on the second day, a scratch was generated with a scratching instrument. The plate was then gently rinsed 2-3 times with PBS, followed by addition of medium containing 1% FBS and incubation at 37°C with 5% CO_2_. Celigo scanning was performed at three time points based on the extent of healing. Migration area analysis was performed using Celigo.

### Transwell migration assay

For the transwell migration assay, a serum-free cell suspension was prepared and added at a cell count of 10^5^ cells per well in a 24-well plate based on preliminary results. Totally 100 µL of the cell suspension was added to the superior compartment, and 600 µL of medium with 30% FBS to the inferior compartment. Upon incubating cells for 24 h at 37°C, fixation was carried out with paraformaldehyde for 15 min, following by a 10-min crystal violet staining. After removing the cells remaining on the superior surface of the membrane, those on the bottom surface were analyzed using a Leica microscope. To assess the migratory ability, migrated cells in four random fields were counted.

### Transwell invasion assay

The transwell invasion assay was conducted following the same procedures as the migration assay. Resuspended cells were adjusted for density accordingly. Following Matrigel rehydration, the chambers were placed in fresh Transwell plates. Then, the cell suspension (200 µL) and medium containing 30% FBS (750 µL) were added to the superior and inferior compartments, respectively. Upon incubation at 37°C, non-invasive cells were then removed from the superior surface of the chamber using cotton swabs. The cells that traversed the membrane subsequently underwent fixation and staining according to the migration assay section. A Leica microscope was employed for analysis. To assess the invasive capability, invasive cells in four random fields were counted.

### Statistical analysis

Experimental data were expressed as mean±SEM, with each experiment carried out independently at least thrice. GraphPad Prism 10.0 was employed for data analysis. The t-test and one-way ANOVA were conducted for group pair and multiple group comparisons, respectively. *P*<0.05 reflected statistical significance.

## Results

### COA6 is upregulated in breast cancer tissues

**Figure 1:**
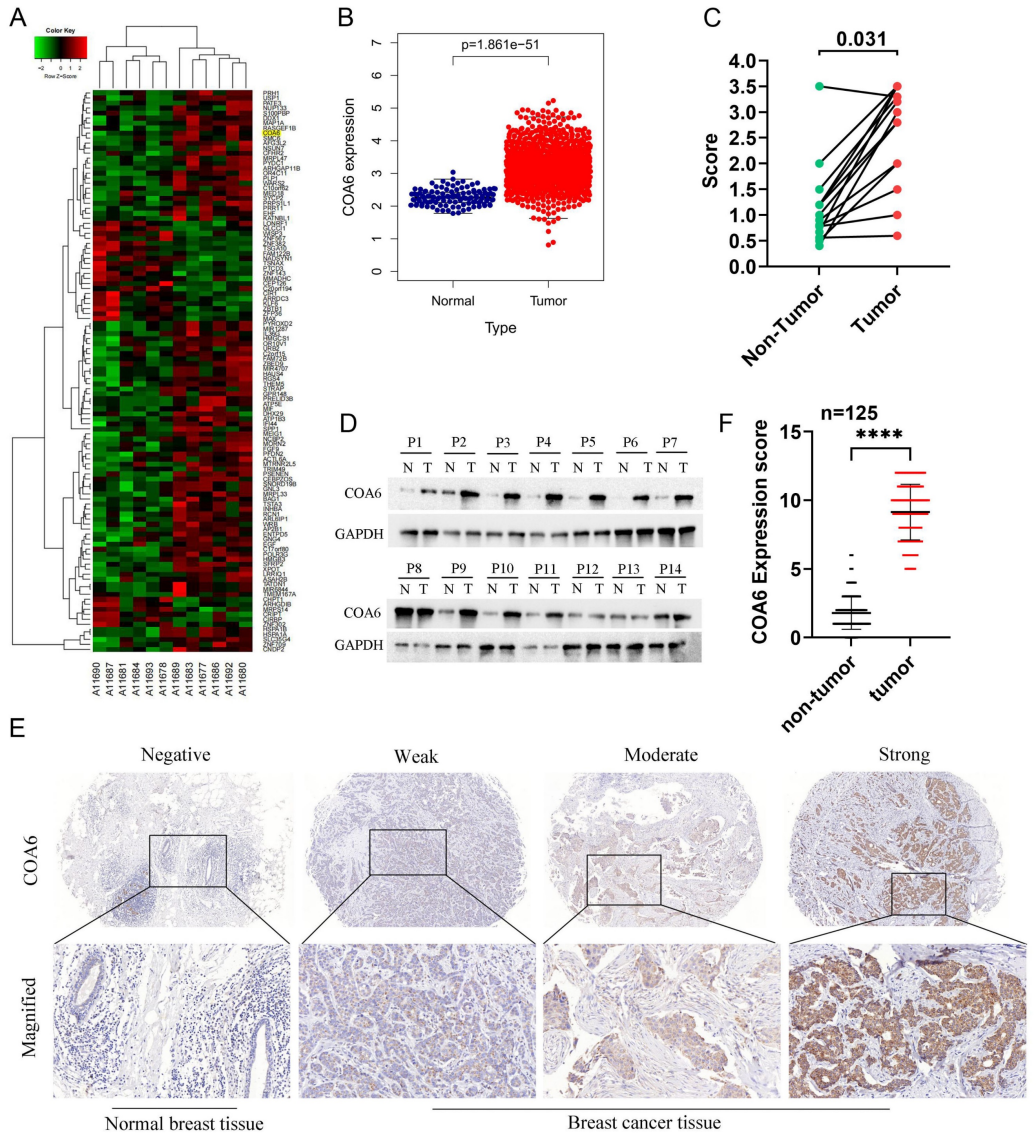
**COA6 is upregulated in breast cancer tissues.** (A) Transcriptome sequencing was carried out in 6 pairs of breast cancer and adjacent noncancerous breast tissue specimens; representative differential genes are shown. (B) COA6 mRNA expression in breast cancer in TCGA. (C) COA6 mRNA levels and (D) protein amounts in 14 pairs of breast cancer and matched paracancerous specimens by qRT-PCR and immunoblot, respectively. (E) Representative IHC micrographs for COA6 detection in human breast cancer paraffin sections. (F) COA6 expression determined by tissue microarray in cancerous and benign histological specimens (n=125).

To identify the key factors involved in OXPHOS-mediated breast cancer, transcriptome sequencing was performed to assess six matched pairs of breast cancer and adjacent noncancerous breast tissue samples. The differential gene expression analysis revealed significant overexpression of COA6 mRNA in breast cancer tissue specimens (Figure 1A). Subsequent assessment of the TCGA database using DESeq2/edgeR confirmed a significant upregulation of COA6 in the breast cancer group. Statistically significant differences were detected (Figure 1B). Consistent with these findings, qRT-PCR and immunoblot analyses of 14 matched pairs of breast cancer and adjacent noncancerous tissue samples demonstrated significantly higher COA6 expression levels in breast cancer tissues (Figure 1C and 1D). IHC staining was performed to assess the clinical significance of COA6 in 125 paired cancerous and benign histological samples. COA6 was mainly localized to the cytoplasm and highly expressed in cancerous tissues in comparison with benign controls (Figure 1E and 1F).

### Correlation of COA6 expression with clinicopathological variables in breast cancer

**Figure 2:**
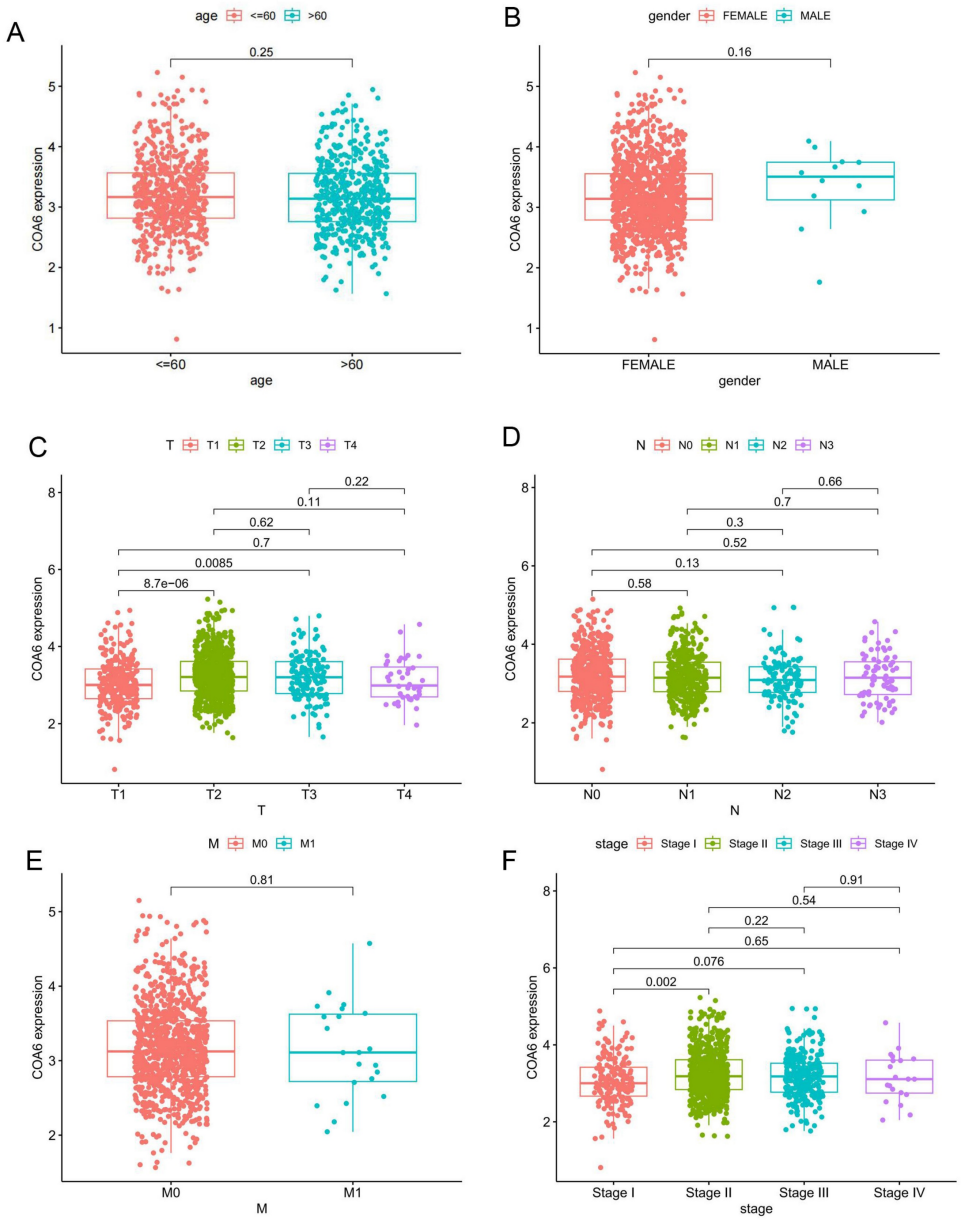
** Associations of COA6 expression with clinicopathological indexes in breast cancer.** (A) Age. (B) Gender. (C) T (tumor size) stage. (D) N (lymph node metastasis) stage. (E) M (distant metastasis) stage. (F) Tumor stage (tumor size, lymph node metastasis, and distant metastasis). Independent samples t-test was used to compare the means of two groups.

Out of the 125 breast cancer cases studied, 15 (12%) exhibited low COA6 expression, while 110 (88%) showed high COA6 expression. Both groups were similar in age, vascular tumor thrombus, lymph node metastasis, tumor differentiation, prognosis, and receptor expression (*p*>0.05). However, tumor size and histological grade were markedly different (*p*<0.05) (Table 1). In a subsequent analysis of COA6 gene expression in distinct clinical subgroups of breast cancer cases in TCGA, no significant differences were detected in COA6 mRNA levels across ages, genders, N (lymph node metastasis) stages, and M (distant metastasis) stages (Figure 2A, 2B, 2D and 2E). However, based on T (tumor size) stage, COA6 mRNA amounts were significantly elevated in the T2 and T3 groups compared with T1 cases (*p*<0.05) (Figure 2C). Furthermore, when considering tumor stage that combines tumor size, lymph node metastasis, and distant metastasis, COA6 mRNA amounts were elevated in stage II cases compared with the stage I group (*p*<0.05) (Figure 2F). The above findings indicate a possible correlation between increased COA6 levels and breast cancer progression.

### Prognostic significance of COA6 expression in breast cancer

**Figure 3:**
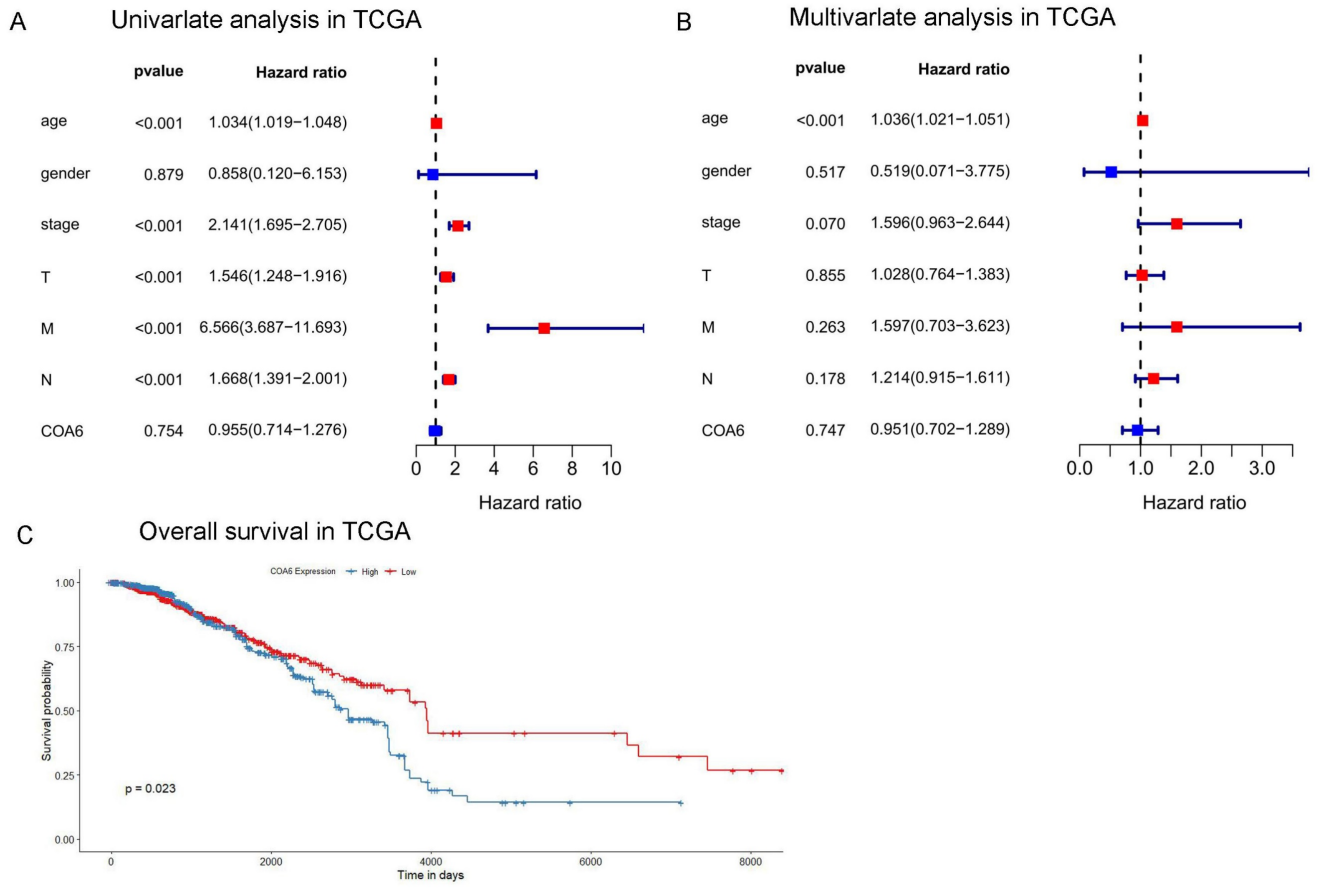
**Effect of COA6 on patient prognosis in breast cancer.** (A) Univariate and (B) multivariate Cox regression analyses in TCGA. (C) Kaplan-Meier curve analysis of the effect of COA6 on overall survival in breast cancer patients of TCGA.

To examine COA6's prognostic significance in breast cancer, a bioinformatic analysis was performed for TCGA datasets. Univariate Cox regression analysis revealed that age, tumor stage, T stage, N stage, and M stage were independent unfavorable prognostic factors of breast cancer. Furthermore, multivariate Cox regression analysis showed age represented an independent prognostic factor (Figure 3A and 3B). In survival analysis, cases with increased COA6 levels displayed reduced overall survival (OS) (Figure 3C). This finding highlights the potential prognostic significance of COA6 in breast cancer.

### COA6 expression is enhanced in cultured breast cancer cells

**Figure 4:**
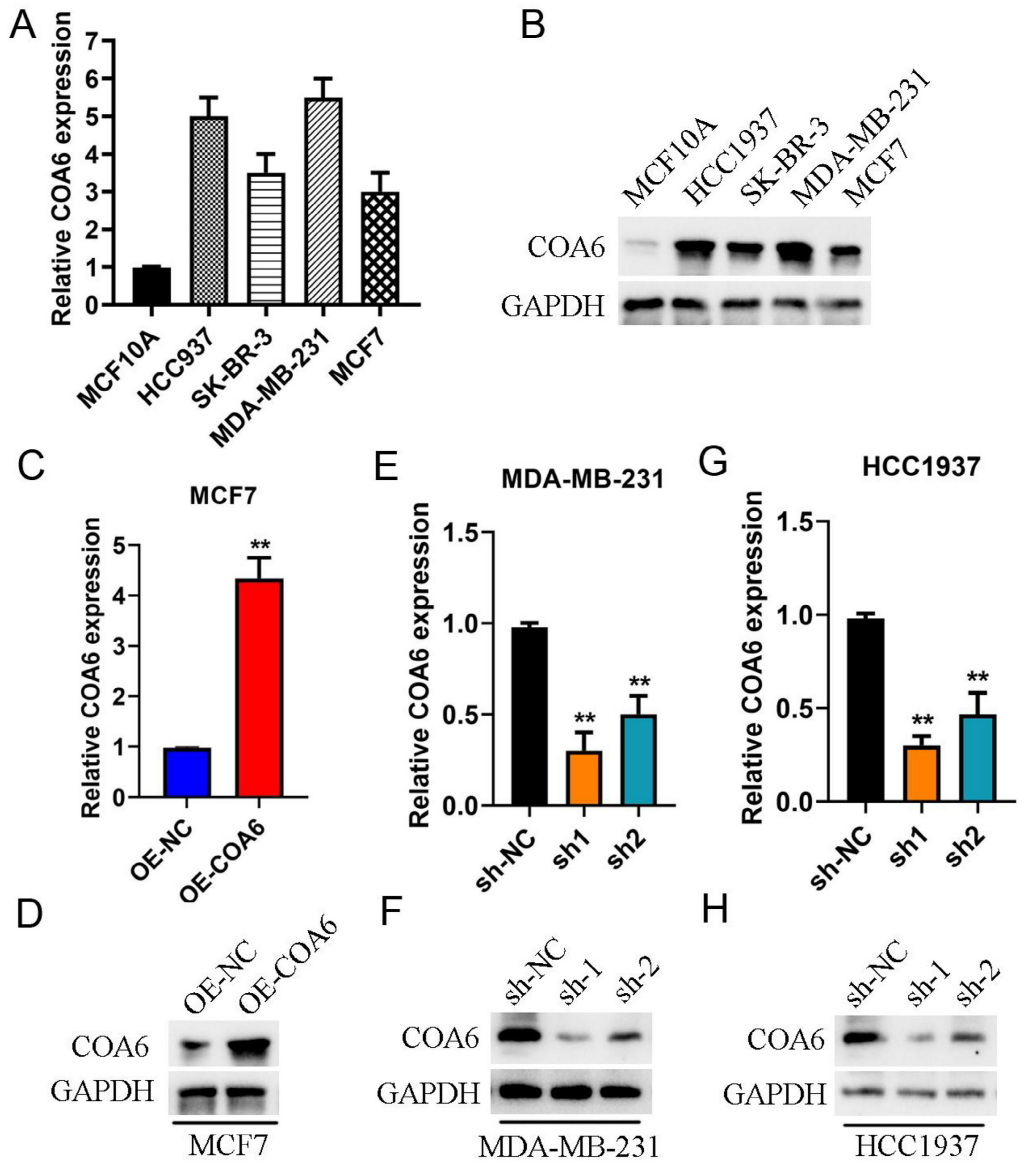
**COA6 expression in breast cancer cells.** (A) qRT-PCR detection of COA6 mRNA in four breast cancer and one noncancerous breast epithelial cell lines. (B) Immunoblot detection of the COA6 protein in breast cancer and normal breast ductal epithelial cells. (C) qRT-PCR and (D) immunoblot analyses of COA6 in MCF-7 cells with or without stable overexpression of COA6. (E) qRT-PCR and (F) immunoblot analyses of COA6 in MDA-MB-231 cells with COA6 knockdown. (G) qRT-PCR and (H) immunoblot analyses of COA6 in HCC1937 cells with COA6 knockdown. **p<0.01.

COA6 mRNA and protein amounts were assessed in MDA-MB-231, HCC1937, SK-BR-3 and MCF-7 (breast cancer cells) comparatively to the noncancerous breast ductal epithelial MCF10A cell line using qRT-PCR (Figure 4A) and immunoblot (Figure 4B). The data revealed elevated COA6 mRNA and protein amounts in breast cancer cells. Specifically, MDA-MB-231 cells exhibited the highest expression, while MCF7 cells showed the lowest values. In subsequent experiments, MCF-7 cells after transfection with a lentivirus overexpressing COA6 showed a significant increase in COA6 expression compared with control lentivirus-transfected cells (Figure 4C and 4D). Conversely, transfection with shCOA6 lentivirus resulted in markedly downregulated COA6 in MDA-MB-231 and HCC1937 cells in comparison with the respective shNC control lentivirus-transfected counterparts (Figure 4E, 4F, 4G and 4H).

### COA6 enhances breast cancer cell proliferation

**Figure 5:**
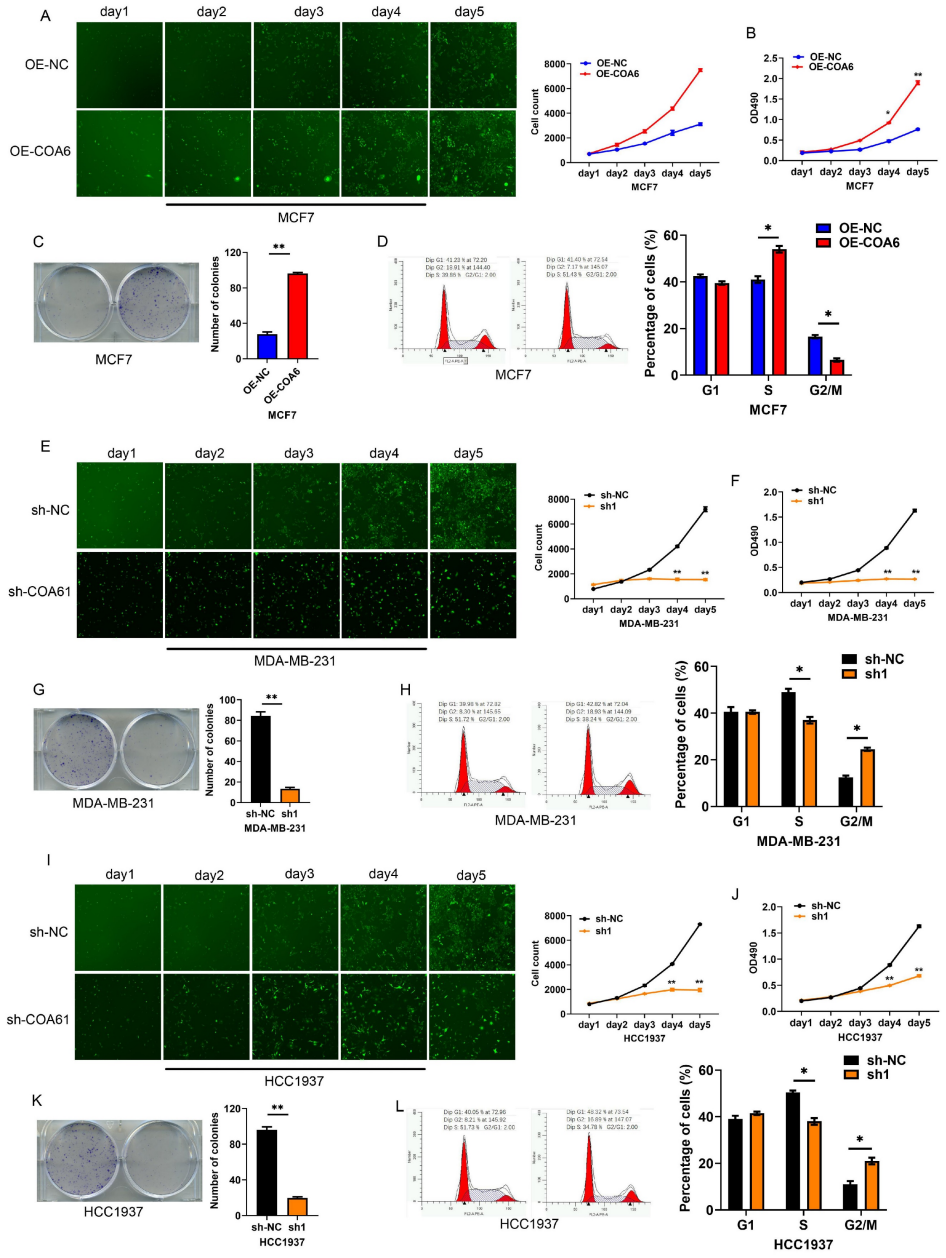
**COA6 enhances breast cancer cell proliferation.** (A) Cell counts were compared by Celigo analysis at 5 d in COA6-overexpressing MCF-7 and control cells. (B) MTT assay, (C) Colony formation assay and (D) Cell cycle analysis of MCF-7 cells with or without stable overexpression of COA6. (E) Celigo analysis, (F) MTT assay, (G) Colony formation assay and (H) Cell cycle analysis of cell viability in MDA-MB-231 cells with or without COA6 knockdown. (I) Celigo analysis, (J) MTT assay, (K) Colony formation assay and (L) Cell cycle analysis of cell viability in HCC1937 cells with or without COA6 knockdown. *p<0.05, **p<0.01.

The Celigo assay was employed to investigate COA6's effect on breast cancer cell proliferation. The obtained growth curves showed that COA6 overexpression increased viability in MCF7 cells, while COA6 knockdown decreased cell viability in both MDA-MB-231 and HCC1937 cells (Figure 5A, 5E, and 5I). The MTT assay confirmed that cell viability was enhanced after COA6 overexpression in MCF7 cells and reduced after transfection with shCOA6 in MDA-MB-231 and HCC1937 cells (Figure 5B, 5F, and 5J). Furthermore, the clonogenic assay demonstrated that COA6 upregulation increased the number of cell colonies, while COA6 downregulation had the opposite effect (Figure 5C, 5G, and 5K). Cell cycle analysis showed that high COA6 expression promoted the transition from G1 to S, thereby enhancing cell cycle progression in MCF7 cells (Figure 5D). Conversely, COA6 knockdown resulted in cell cycle arrest (Figure 5H and 5L). These results demonstrate that COA6 enhances the proliferative ability of breast cancer cells.

### COA6 enhances the migratory and invasive capabilities of breast cancer cells

**Figure 6:**
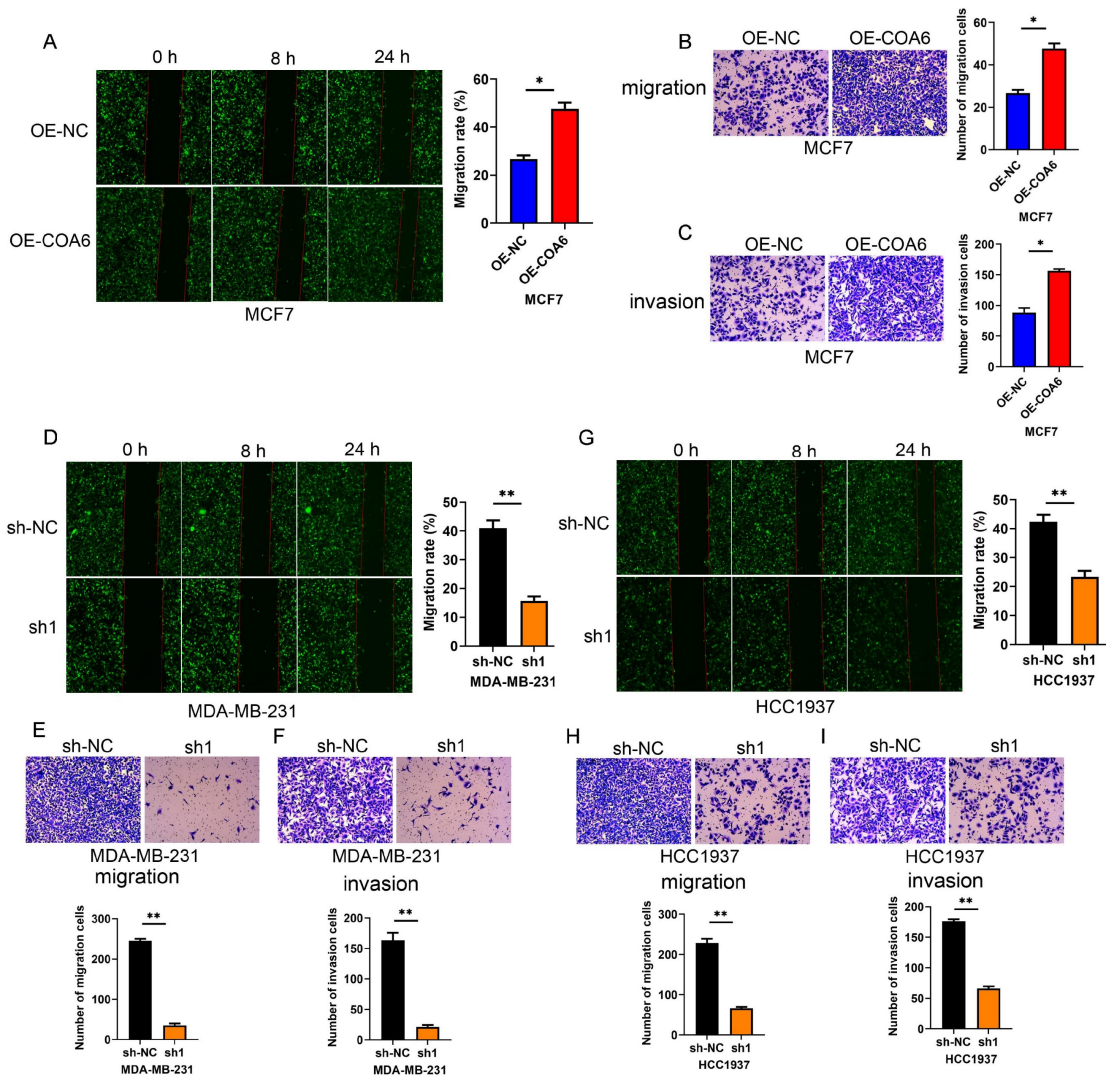
**COA6 enhances migration and invasion in breast cancer cells.** (A) Wound healing, (B) Transwell migration (C) and Transwell invasion assays were carried out in COA6-overexpressing MCF7 cells. (D) Wound healing, (E) Transwell migration and (F) Transwell invasion assays were performed to detect cell migration and invasion after COA6 knockdown in MDA-MB-231 cells. (G) Wound healing, (H) Transwell migration and (I) Transwell invasion assays were carried out to detect cell migration and invasion after COA6 knockdown in HCC1937 cells. *p<0.05, **p<0.01.

COA6's effects on the migratory and invasive capabilities of cells were examined by the wound healing assay. The data showed that COA6 overexpression enhanced the wound recovery capacity of MCF7 cells (Figure 6A). Conversely, the wound recovery abilities of MDA-MB-231 and HCC1937 cells were reduced after COA6 suppression (Figure 6D and 6G). These findings were supported by Transwell assays in which COA6 upregulation increased the number of migratory MCF7 cells, while COA6 silencing in MDA-MB-231 and HCC1937 inhibited cell migration (Figure 6B, 6E, and 6H). Furthermore, cell invasion was examined with Transwell chambers coated with Matrigel. The results indicated that COA6 overexpression promoted cell invasion, which was attenuated in both MDA-MB-231 and HCC1937 cells upon COA6 knockdown (Figure 6C, 6F, and 6I). Therefore, COA6 increases the migratory and invasive capabilities of breast cancer cells.

### COA6 attenuates breast cancer cell apoptosis

**Figure 7:**
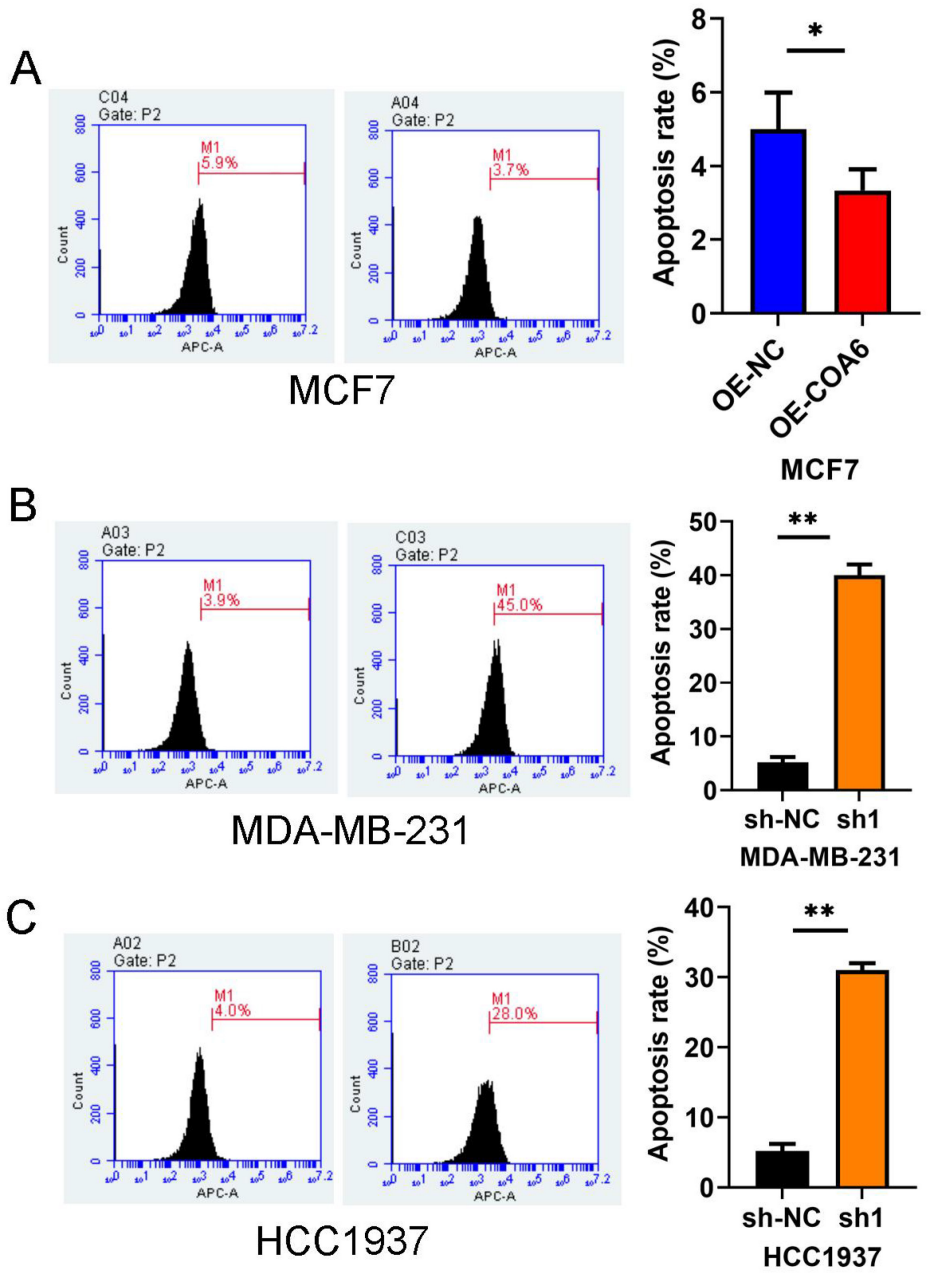
**COA6 attenuates apoptosis in breast cancer cells.** (A) Flow cytometry was performed with an Annexin V FITC kit to quantitate apoptosis in MCF7 cells after COA6 overexpression. The apoptosis rates of COA6-silenced MDA-MB-231 cells (B) and HCC1937 cells (C) were determined. *p<0.05, **p<0.01.

COA6's effect on cell apoptosis was examined flow-cytometrically. The experimental data revealed markedly reduced apoptosis (by approximately 3.7%) in MCF7 cells after COA6 overexpression (Figure 7A). Conversely, in COA6-silenced cells, apoptosis was starkly increased, by 45% and 28% in MDA-MB-231 and HCC1937 cells, respectively, versus the respective control groups (Figure 7B and 7C).

### COA6's impact on OXPHOS

**Figure 8:**
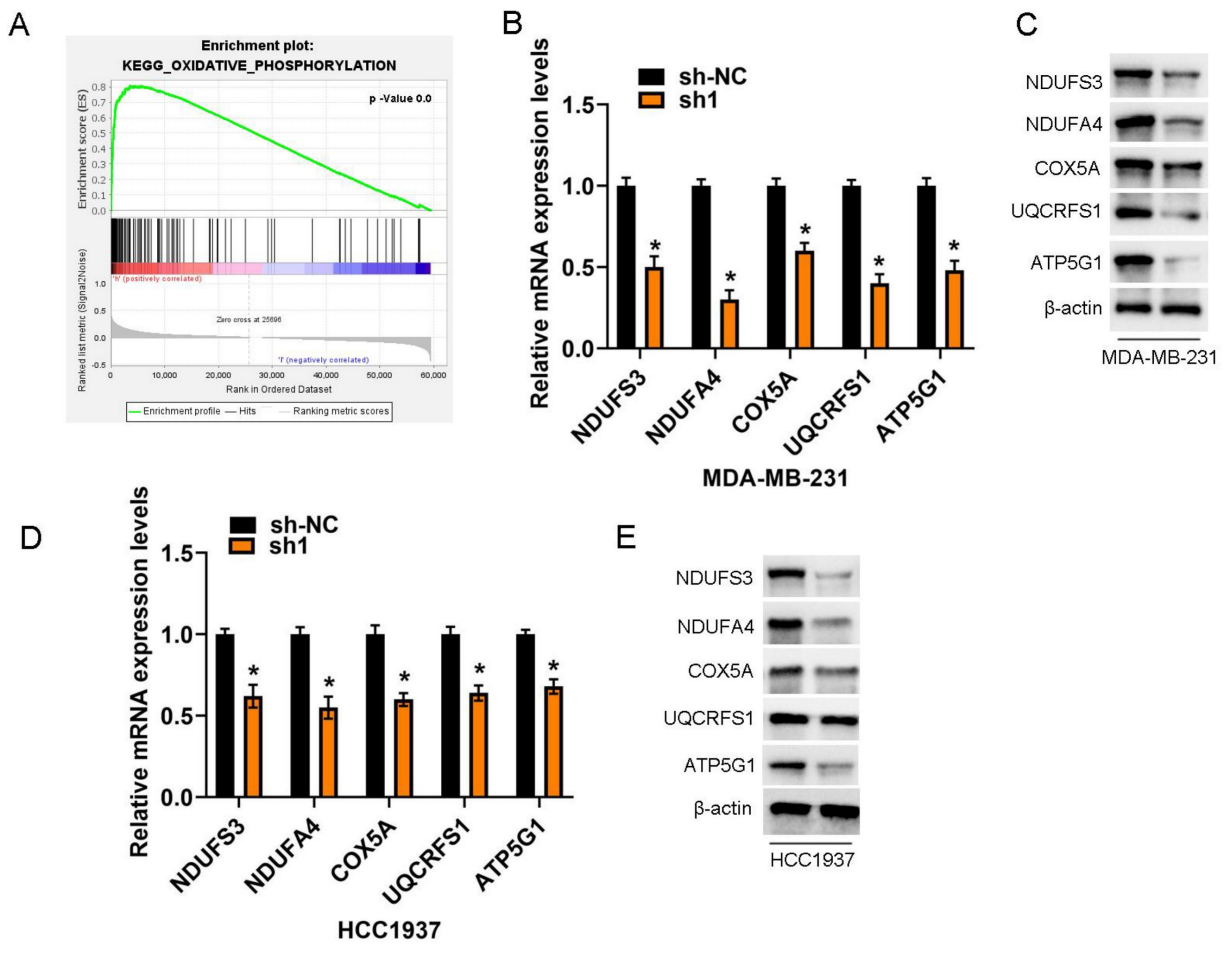
**COA6 regulates OXPHOS metabolism.** (A) KEGG pathway enrichment analysis of genes associated with COA6. (B) qRT-PCR analysis of the mRNA expression of representative subunits of the OXPHOS complex in MDA-MB-231 cells after COA6 knockdown. (C) Immunoblot detection of protein expression levels in MDA-MB-231 cells. (D) qRT-PCR analysis of the mRNA expression of representative subunits of the OXPHOS complex in HCC1937 cells after COA6 knockdown. (E) Immunoblot detection of protein expression levels in HCC1937 cells. *p<0.05.

KEGG pathway enrichment analysis revealed a significant enrichment of differentially expressed genes with positive correlations with COA6 in OXPHOS signaling among breast cancer cases. This observation underscores the critical importance of OXPHOS research in breast cancer (Figure 8A). Stable knockdown of COA6 in MDA-MB-231 and HCC1937 cells starkly lowered the mRNA expression of key subunits of the OXPHOS complex, including Complexes I (NDUFA3, NDUFA4), III (UQCRFS1), IV (COX5A), and V (ATP5G1) (Figure 8B and 8D). These findings were confirmed by immunoblot, which also verified changes in the protein expression levels of the above subunits (Figure 8C and 8E).

## Discussion

The current study revealed firstly that COA6 is significantly overexpressed in breast cancer tissues. This was evidenced by transcriptome sequencing, TCGA-BRCA dataset analysis, and clinical sample analysis. COA6 overexpression was associated with clinicopathological parameters and unfavorable prognosis in breast cancer patients. These findings prompted us to further examine COA6's function in breast cancer progression.

The COA6 gene, located at 1q42, spans approximately 10.59 kb and comprises three exons and two introns. The latter exons encode 155 amino acids 14. Previous studies have reported a tight association of COA6 with electron transfer during OXPHOS. The OXPHOS electron transport chain comprises five complexes, among which complex IV, also known as cytochrome c oxidase (CcO), requires copper atoms for structural stabilization and function 15. COA6, a copper chaperone, is responsible for delivering copper to a subunit of CcO, particularly cytochrome c oxidase subunit 2 (COX2) 16. Recent evidence reveals COA6 also interacts with other copper metal chaperones, including SCO1 and SCO2, and overlaps functionally with SCO2 in the biogenesis of the CuA site 17,18. Abnormalities in COA6 may impair CcO assembly, resulting in decreased activity and causing multiple mitochondrial diseases. Mutations in COA6 have been implicated in combined deficiency of mitochondrial complexes I and IV in myocardial tissue, highlighting a crucial role for COA6 in electron transfer during OXPHOS 19,20. However, how COA6 functions and interacts with other partners in CuA site maturation remain mostly unexplored.

Recent studies have indicated COA6 may act as an oncogene in liver hepatocellular carcinoma (LIHC), contributing to the molecular mechanisms of cuproptosis and immune cell expression. COA6 was identified as a potential immunotherapeutic target and prognostic predictor in LIHC 21. Similarly, COA6 upregulation was detected in hepatocellular carcinoma (HCC) tissues, with significantly associations with poor survival outcomes 22. In lung adenocarcinoma, high COA6 expression was linked to unfavorable prognosis and enhanced OXPHOS 23. In prostate cancer, high COA6 expression enhanced OXPHOS and promoted tumor progression 12. The current research aligns with the above findings, demonstrating COA6 is significantly overexpressed in breast cancer and has direct associations with clinicopathological parameters and unfavorable prognosis. The above *in vitro* data further supports the oncogenic role of COA6, suggesting its overexpression enhances the proliferative, migratory, and invasive properties of breast cancer cells while attenuating apoptosis. These findings collectively underscore the promoting effects of COA6 in various cancers.

The Warburg effect originally considered glycolysis as the primary metabolic pathway in tumorigenesis and cancer development, with OXPHOS considered to be less critical 24. However, recent evidence challenges this notion, revealing OXPHOS upregulation in some cancer cells 25. Contemporary studies highlight the importance of oxygenation in ATP production for specific cancers, which may be detected by genetic biomarkers such as SMARCA4 mutations 26. Myc-driven triple-negative breast cancer and other malignant diseases heavily rely on mitochondrial fatty acid oxidation for sustained growth 27. Pancreatic cancer is an example of a highly expressing OXPHOS tumor, exhibiting robust expression of mitochondrial respiratory complex I at both protein and mRNA levels. Complex I inhibitors were shown to be effective in combination with conventional chemotherapy 28. Additionally, melanomas with elevated PGC1α expression showed increased mitochondrial capacity 29. In lung cancer, tumor tissue samples have elevated glucose oxidation rate and tricarboxylic acid cycle activity compared with adjacent noncancerous tissue specimens 30. The above findings revealed significant associations of numerous genes positively linked to COA6 with the OXPHOS gene set, indicating a close relationship between COA6 and OXPHOS in breast cancer. The high-COA6 expression group showed significant activation of the OXPHOS pathway. This may explain the associations of COA6 expression with tumor stage and poorer overall survival in patients with elevated COA6 levels. Additionally, this study confirms that depleted COA6 downregulates key subunits of the OXPHOS complex in breast cancer cells, thereby impeding cancer cell proliferation.

In summary, the current findings highlight a close connection between increased COA6 levels and enhanced OXPHOS activity. COA6 has significant effects on breast cancer, and decreasing its levels may reduce OXPHOS, ultimately inhibiting breast cancer cells. Further clinical investigation is warranted to provide substantial insights into the role of COA6 in mitochondrial metabolism, particularly in breast cancer. However, this work unveils an oncogenic role for COA6 in breast cancer.

## Funding

The current work was supported by the Foundation of Nantong Science and Technology Bureau [MS22022015], and the Nantong Municipal Health Committee Project [MS2023062].

## Figures and Tables

**Table 1:** Relationship between the expression of COA6 and clinicopathological data.

Parameters	Total n (%)	Low expression n (%)	High expression n (%)	*p*
Age				
≤50	65 (52)	8 (6.4)	57 (45.6)	0.750
>50	60 (48)	7 (5.6)	53 (42.4)	
Tumor size (cm)				
≤3	73 (58.4)	14 (11.2)	59 (47.2)	0.041^a^
>3	52 (41.6)	1 (0.8)	51 (40.8)	
Vascular tumor thrombus				
Present	42 (33.6)	4 (3.2)	38 (30.4)	0.630
Absent	83 (66.4)	11 (8.8)	72 (57.6)	
Lymph node metastasis				
Present	70 (56)	7 (5.6)	63 (50.4)	0.240
Absent	55 (44)	8 (6.4)	47 (37.6)	
Tumor differentiation				
High differentiation	16 (12.8)	7 (5.6)	9 (7.2)	
Middle differentiation	59 (47.2)	4 (3.2)	55 (44)	0.038^a^
Low differentiation	50 (40)	4 (3.2)	46 (36.8)	
Prognosis				
Survive	117 (93.6)	14 (11.2)	103 (82.4)	0.780
Death	8 (6.4)	1(0.8)	7 (5.6)	
Receptor expression				
Triple negative	23 (18.4)	2 (1.6)	21 (16.8)	
Triple positive	14 (11.2)	6 (4.8)	8 (6.4)	0.570
HR+/HER2-	65 (52)	6 (4.8)	59 (47.2)	
HR-/HER2+	23 (18.4)	1 (0.8)	22(17.6)	

## Abbreviations

OXPHOS: Oxidative phosphorylation
COA6: Cytochrome c oxidase assembly factor 6
OS: Overall survival
TCGA: The Cancer Genome Atlas
IHC: Immunohistochemical

## References

[1] Xia C, Dong X, Li H (2022). Cancer statistics in China and United States, 2022: profiles, trends, and determinants. Chin Med J (Engl).

[2] Domínguez-Cejudo MA, Gil-Torralvo A, Cejuela M (2023). Targeting the Tumor Microenvironment in Breast Cancer: Prognostic and Predictive Significance and Therapeutic Opportunities. Int J Mol Sci.

[3] Vaupel P, Multhoff G (2021). Revisiting the Warburg effect: historical dogma versus current understanding. J Physiol.

[4] Ghosh P, Vidal C, Dey S (2020). Mitochondria Targeting as an Effective Strategy for Cancer Therapy. Int J Mol Sci.

[5] Ashton TM, McKenna WG, Kunz-Schughart LA (2018). Oxidative phosphorylation as an emerging target in cancer therapy. Clin Cancer Res.

[6] Gong Y, Ji P, Yang YS (2021). Metabolic-Pathway-Based Subtyping of Triple- Negative Breast Cancer Reveals Potential Therapeutic Targets. Cell Metab.

[7] Maghool S, G Cooray ND, Stroud DA (2019). Structural and functional characterization of the mitochondrial complex IV assembly factor Coa6. Life Sci Alliance.

[8] Pacheu-Grau D, Wasilewski M, Oeljeklaus S (2020). COA6 Facilitates Cytochrome c Oxidase Biogenesis as Thiol- reductase for Copper Metallochaperones in Mitochondria. J Mol Biol.

[9] Swaminathan AB, Gohil VM (2022). The Role of COA6 in the Mitochondrial Copper Delivery Pathway to Cytochrome c Oxidase. Biomolecules.

[10] Soma S, Morgada MN, Naik MT (2019). COA6 Is Structurally Tuned to Function as a Thiol-Disulfide Oxidoreductase in Copper Delivery to Mitochondrial Cytochrome c Oxidase. Cell Rep.

[11] Baertling F, van den Brand MAM, Hertecant JL (2015). Mutations in COA6 cause cytochrome c oxidase deficiency and neonatal hypertrophic cardiomyopathy. Hum Mutat.

[12] Zahalka AH, Arnal-Estapé A, Maryanovich M (2017). Adrenergic nerves activate an angio-metabolic switch in prostate cancer. Science.

[13] Zhang X, Dong W, Zhang J (2021). A Novel Mitochondrial-Related Nuclear Gene Signature Predicts Overall Survival of Lung Adenocarcinoma Patients. Front Cell Dev Biol.

[14] Stroud DA, Maher MJ, Lindau C (2015). COA6 is a mitochondrial complex IV assembly factor critical for biogenesis of mtDNA-encoded COX2. Hum Mol Genet.

[15] Pacheu-Grau D, Bareth B, Dudek J (2015). Cooperation between COA6 and SCO2 in COX2 maturation during cytochrome c oxidase assembly links two mitochondrialcardiomyopathies. Cell Metab.

[16] Bi R, Zhang W, Zhang DF (2018). Genetic association of the cytochrome c oxidase-related genes with Alzheimer's disease in Han Chinese. Neuropsychopharmacology.

[17] Brischigliaro M, Zeviani M (2021). Cytochrome c oxidase deficiency. Biochim Biophys Acta Bioenerg.

[18] Visuttijai K, Hedberg-Oldfors C, Lindgren U (2021). Progressive external ophthalmoplegia associated with novel MT-TN mutations. Acta Neurol Scand.

[19] Wintjes LTM, Kava M, van den Brandt FA (2021). A novel variant in COX16 causes cytochrome c oxidase deficiency, severe fatal neonatal lactic acidosis, encephalopathy, cardiomyopathy, and liver dysfunction. Hum Mutat.

[20] Ghosh A, Trivedi PP, Timbalia SA (2014). Copper supplementation restores cytochrome c oxidase assembly defect in a mitochondrial disease model of COA6 deficiency. Hum Mol Genet.

[21] Han S, Ye T, Mao Y (2023). Cuproptosis-Related Genes CDK1 and COA6 Involved in the Prognosis Prediction of Liver Hepatocellular Carcinoma. Dis Markers.

[22] Zhao X, Chen J, Yin S (2022). The expression of cuproptosis-related genes in hepatocellular carcinoma and their relationships with prognosis. Front Oncol.

[23] Zhang M, Liao X, Ji G (2023). High Expression of COA6 Is Related to Unfavorable Prognosis and Enhanced Oxidative Phosphorylation in Lung Adenocarcinoma. Int J Mol Sci.

[24] WARBURG O (1958). Photosynthesis. Science.

[25] Gaude Ed, Frezza C (2016). Tissue-specific and convergent metabolic transformation of cancer correlates with metastatic potential and patient survival. Nat Commun.

[26] Lissanu Deribe Y, Sun Y, Terranova C (2018). Mutations in the SWI/SNF complex induce a targetable dependence on oxidative phosphorylation in lung cancer. Nat Med.

[27] Camarda R, Zhou AY, Kohnz RA (2016). Inhibition of fatty acid oxidation as a therapy for MYC-overexpressing triplenegative breast cancer. Nat Med.

[28] Masoud R, Reyes-Castellanos G, Lac S (2020). Targeting mitochondrial complex I overcomes chemoresistance in high OXPHOS pancreatic Cancer. Cell Rep Med.

[29] Vazquez F, Lim JH, Chim H (2013). PGC1α expression defines a subset of human melanoma tumors with increased mitochondrial capacity and resistance to oxidative stress. Cancer Cell.

[30] Bluemel G, Planque M, Madreiter-Sokolowski CT (2021). PCK2 opposes mitochondrial respiration and maintains the redox balance in starved lung cancer cells. Free Radic Biol Med.

